# A Review for Solitary Plasmacytoma of Bone and Extramedullary Plasmacytoma

**DOI:** 10.1100/2012/895765

**Published:** 2012-05-02

**Authors:** Sevil Kilciksiz, Omur Karakoyun-Celik, Fulya Yaman Agaoglu, Ayfer Haydaroglu

**Affiliations:** ^1^Radiation Oncology Clinic, Okmeydani Training and Research Hospital, Ministry of Health, Istanbul, Turkey; ^2^Department of Radiation Oncology, Faculty of Medicine, Celal Bayar University, Manisa, Turkey; ^3^Department of Radiation Oncology, Oncology Institute, Istanbul University, Istanbul, Turkey; ^4^Department of Radiation Oncology, Faculty of Medicine, Ege University, Izmir, Turkey

## Abstract

Solitary plasmacytoma (SP) is characterized by a mass of neoplastic monoclonal plasma cells in either bone (SBP) or soft tissue without evidence of systemic disease attributing to myeloma. Biopsy confirmation of a monoclonal plasma cell infiltration from a single site is required for diagnosis. The common presentation of SBP is in the axial skeleton, whereas the extramedullary plasmacytoma (EMP) is usually seen in the head and neck. The ratio of SP seen at males to females is 2 : 1 and the median age of patients is 55 years. The incidence rate of SP in black race is approximately 30% higher than the white race. Incidence rate increases exponentially by advancing age. SBP has a significant higher risk for progression to myeloma, and the choice of treatment is radiotherapy (RT) that is applied with curative intent at min. 4000 cGy. By only RT application, long-term disease-free survival (DFS) is possible for approximately 30% of patients with SBP and 65% of patients with EMP.

## 1. Background

The solitary plasmacytoma (SP) is characterized by a localized accumulation of neoplastic monoclonal plasma cells without a proof of a systemic plasma cell proliferative disorder. It is an infrequent form of plasma cell neoplasm and represents 5% to 10% of all plasma cell neoplasms according to the literature [1–5]. It can be classified into 2 groups regarding to location; defined as solitary plasmacytoma of the bone (SBP) and extramedullary plasmacytoma (EMP) [1, 6]. SP mostly occurs in the bones of the axial skeleton, such as vertebra and skull [1, 2]. The EMP is generally observed in the head and neck and most frequently in the nasal cavity and nasopharynx [5, 7, 8]. The median age of the patients with either SBP or EMP is 55 years [1]. The male-to-female ratio of SP is 2 : 1 [5, 7–10]. Incidence rate rises exponentially by advancing age; however, it is less prominent at older ages in comparison with multiple myeloma (MM) [11, 12]. The incidence rate of SP in black race is around 30% higher than white race [11, 12].

## 2. Diagnosis and Staging

The differentiation criteria for both SBP and EMP from myeloma is lack of CRAB (increased calcium, renal insufficiency, anemia, or multiple bone lesions) features. Diagnostic analysis consists of history, physical examination, complete blood count, bone marrow biopsy, serum protein electrophoresis, evaluation of the urine for myeloma protein, and skeletal survey. The diagnosis of SBP requires solitary bone lesion confirmed by skeletal survey, plasma cell infiltration proven by biopsy, normal bone marrow biopsy (<10% plasma cells), and lack of myeloma-related organ dysfunction [13]. Diagnostic criteria of extramedullary plasmacytoma (EMP) are tissue biopsy-indicating monoclonal plasma cell histology, bone marrow plasma cell infiltration less than 5% of all nucleated cells, absence of osteolytic bone lesions or other tissue involvement without proof of myeloma, hypercalcemia or renal failure, and low-serum M protein concentration, if exists [8, 9].

According to the Durie and Salmon staging system, solitary bone plasmacytomas are regarded as stage I myeloma [13]. Therefore, stage I myeloma contains all of the following criteria: hemoglobin > 10 g/dL, normal level of serum calcium, normal bone structure or solitary plasmacytoma only, and low M-component (IgG < 5 g/dL, IgA < 3 g/dL, urine light chains < 4 g/24 h) [12, 13].

Changes in laboratory results of secretory plasmacytoma generally indicate immunoglobulin production, blood calcium level alterations, kidney dysfunction, and elevated serum *β*-2-microglobulin levels. Moreover, secretory plasmacytoma may be associated with POEMS syndrome (Polyneuropathy, Organomegaly, Endocrinopathy, Multiple myeloma, and Skin changes) [14].

Skeletal survey is beneficial to detect osteoblastic response to bone destruction. Nevertheless, computed tomography (CT) may be more helpful to detect the extent of bone destruction, and magnetic resonance imaging (MRI) may also be helpful to determine the multiple vertebra lesions or bone marrow disease [15]. The lesions are darker or isointense on T1-weighted images and hyperintense on T2-weighted images and contrast enhanced [16]. Upon the detection of multiple vertebra lesions or positivity on 18F-fluorodeoxyglucose positron emission tomography (FDG-PET) or bone marrow disease, some patients having been suspected SPB will be upstaged for MM [17, 18].

## 3. Prognostic Factors

There are three patterns of failure, these are development of MM, local recurrence, and development of new bone lesions without MM [19]. In comparison with EMPs, SBPs have poor prognosis [20]. SBP has a significantly higher risk for progression to myeloma at a rate of 65–84% in 10 years and 65–100% in 15 years. In spite of a curative treatment, the median time to progression to MM is 2 to 3 years [20–26]. The 10-year overall survival (OS) rate is 70% in EMP [2, 7–9, 11, 27]. Patients with EMP have improved survival period when compared to patients with SBP that develops into MM in 50–60% of patients [1, 26, 28–30]. Patients with EMP that progressed to MM had a 100% 5-year survival rate as compared to 33% for SBP [31]. In a review of 721 EMP cases by Alexiou et al., after treatment, approximately 65% of the patients had no recurrence and did not progress to MM, whereas 22% experienced recurrence, and 15% of the cases evolved into MM [7].

There are controversial reports with respect to the factors that influence the risk and frequency of progression to MM such as age [3, 20, 21, 23, 24, 28, 32, 33]. In our previous study, younger age was an independent good prognostic factor for progression to MM (The respective hazard ratio was 0.295, *P* = 0.027, 95% CI: 0.100–0.871) [24]. For instance, Knobel et al. analyzed that younger patients, especially with vertebral localization, had the best outcome when treated with moderate dose (≥30 Gy) RT [25].

For SBP, some series have detected that a lesion size of minimum 5 cm, age (e.g., patients aged 40 years and over), spine lesions, RT dose, high M protein levels, existence of light chains, and persistence of M protein after treatment, influence the outcome in these patients and may indicate the presence of higher risks of progression to MM [4, 19, 21, 23, 25, 26, 30, 32–35]. The determination of the prognostic factors of EMPs is complicated by the small number of documented cases and alteration of biological behaviour.

Although the lesion size was reported as the prognostic feature for conversion of SBP into MM, the literature contains conflicts on this matter [4, 21, 26, 32, 33]. In their study, Tsang et al. showed that patients with lesions < 5 cm had a local control rate of 100%, whereas patients with larger tumors had a rate of nearly 40% [32]. In our previous study on solitary plasmacytomas, a statistically significant relation was determined during a univariate analysis between macroscopic tumor existence prior to RT and OS or MMFS. Surgical treatment with RT may be concluded as positive prognostic factor for PFS which may be an indicator for the importance of local tumor bulk for disease progression of patients receiving RT [24].

In most of SBP patients, following RT application, Monoclonal protein is significantly decreased; however, protein disappearance is observed in 20–50% of the patients [1, 26, 35]. In patients with SBP, the disappearance of myeloma protein with involved-field RT predicted long-term DFS and possible treatment [26, 35]. Posttreatment persistent myeloma protein was an adverse prognostic factor, for which adjuvant systemic therapy should be considered [35]. In a study by Wilder et al., the rate of 10-year myeloma-free survival (MMFS) was 91% as compared to 29% in patients with SBP whose M-protein did or did not resolve 1 year after radiation therapy (RT) [35]. On the other hand, in Mayo Clinic experience [23], proof of abnormal serum and/or urine protein was found in 25 out of 46 patients with solitary plasmacytoma of bone. Presence of abnormal proteins even after RT, did not affect both survival and DFS. Local failure has not been observed at the patients who received 45 Gy or more to the solitary lesion.

Many of these findings may indicate the presence of indolent myeloma [1, 19]. Hence, MM may become apparent as soon as the local disease is treated. The new bone lesions, detected as either generalized osteopenia or new abnormalities on MRI studies, may indicate progression to symptomatic MM. The persistence of the M-protein detected following RT or a suppression of the normal immunoglobulin classes may accompany these radiologic abnormalities. In this case, systemic treatment, adjusted for MM, may be applied [21–23, 26, 27].

Pathological factors have been investigated in some studies [22, 36]. It is reported that anaplastic type plasmacytomas, presenting a higher histologic grade, [36] and the existence of a high level of angiogenesis [22] are linked to a poor outcome. Kumar et al. examined whether increased angiogenesis may help to identify the patients likely to progress to myeloma. High-grade angiogenesis was present in 64% of plasmacytomas in their study on plasmacytoma biopsy samples, and these patients were more likely to progress to myeloma and had shorter progression-free survival (PFS) as compared to the patients with low-grade angiogenesis (*P* = .02) [22]. Anaplastic plasmacytomas have some common pathologic and clinical characteristics with aggressive B-cell lymphomas and can develop in the circumstances of immunosuppression and Epstein-Barr virus infection [37].

## 4. Treatment

### 4.1. Radiotherapy

The treatment of SP is largely composed of retrospective studies of small numbers of patients due to its rarity (Tables 1 and 2). Currently, the standard care for SBP is definitely RT. In some cases, surgical intervention may be required for bone instability or for rapidly progressive neurological symptoms like spinal cord compression [12, 38, 39]. The results with surgery alone are not optimal and carry high rates of local recurrence [3].

SBP is a highly radiosensitive disease, for which excellent local control rates (greater than 80%) can be achieved with RT alone [3, 10, 24, 41]. Therefore, RT should be used even after gross total excision to eradicate microscopic residual disease in SBP patients. Even though the optimal dose of RT has not yet been established for SBP, it is recommended that a radiation dose of at least 40 Gy in four weeks is necessary to obtain local control [23, 31, 42]. Mendenhall et al. reported a local control rate of 94% with doses over 40 Gy, which dropped to 64% when patients received less than 40 Gy [31]. In our previous study, lower radiation doses (<50 Gy) were not associated with a significant difference in local control rates; however, they were associated with significantly lower PFS [(HR) 2.279; *P* = 0.044, 95% CI 1.021 to 5.091]. Additionally, approximately 30% of our patients with SBP, who received ≥50 Gy, remained without evidence of any local disease failures [24]. In clinical practice, a radiation dose of 45–50 Gy in 4.5–5 weeks is recommended if allowable by normal tissue tolerances. Although a precise definition of a dose/response relationship has not been clearly demonstrated for SP, preponderance of data suggests that higher doses and treatment with curative-intent required curing the disease, particularly for patients with bulky lesion.

Since the majority of EMP occurs in the head and neck region and radical surgery with curative intent is generally a mutilating procedure, it should be treated with curative-intent RT instead of radical surgery [5, 6, 8, 41]. However, for patients with EMP in other areas, complete surgical removal should be considered. If patients are treated with primary surgery, RT will only be required in case of inadequate surgical margins [7].

In terms of RT-treatment volume definition, using MR imaging for GTV definition offers several advantages over conventional imaging methods, such as staging of the disease and targeting radiation volumes with greater precision due to high soft-tissue contrast and multiplanar imaging [36, 46–48]. It is also excellent for use in follow-up examinations during therapy, as it allows verification of reduction in tumor size [49, 50]. However, CT should be considered in detecting any underlining bone abnormality. Treatment fields should be designed to encompass all diseases observed on MRI and CT scan and should include a margin of healthy tissue. The optimal target volume for RT planning in SPB is controversial. Some authors recommend including the whole bone in the radiation field because of the reported marginal recurrences [28], whereas others recommend including the partial-involved bone [26, 32, 45]. Common practice for radiation fields is to encompass a margin of at least 1.5–2 cm on the tumor shown on MRI as a CTV margin and 5–10 mm as a PTV margin. Prophylactic regional lymph node irradiation is not necessary in SPB, as the regional nodal failure rate is low after local RT without intentional coverage of adjacent nodes [26, 32, 36, 51]. In case of vertebral involvement, fields typically include 1- to 2-uninvolved vertebrae above and below the affected level [38, 52]. The optimal RT target volume is similarly controversial in EMP patients, particularly in head and neck localizations that account for more than 80% of the cases. Addition of elective nodal irradiation to the RT treatment portals provides excellent local control rates. However, it significantly increases the acute and late morbidity [26]. Studies reported excellent nodal control rates as well, without elective nodal irradiation [32, 36, 51]. Only patients with primary tumor localized at Waldeyer's ring received elective nodal irradiation to the first echelon cervical nodes in a study [32]. Due to its morbidity, elective nodal irradiation is not recommended routinely in EMP patients. The RT target volume including cervical lymph nodes is recommended only if clinically involved, or, at high risk, for example, in case of the primary sites involving Waldeyer's ring. Conformal RT using parallel-opposed fields is the most commonly used method to cover the PTV. However, IMRT technique might be considered in some cases to spare the critical structures, such as eyes and salivary glands.

### 4.2. Surgery

For optimal treatment with sufficient local control, a moderate-dose RT combined with surgery (S) is occasionally suggested [9, 20, 21, 28, 32, 36, 53, 54]. Depending on the resectability of the extramedullary lesion, RT combined with surgery may be an acceptable treatment method [8–10, 41]. Despite other studies [51], in our previous study [24], on multivariate analysis, which the median followup was 2.41 years, we found that both a higher dose of radiation and the combination of RT and surgery predicted for better PFS for overall group and SBP subgroup rather than RT alone.

In a review of more than 400 publications between 1905 and 1997, Alexiou et al. reported evidence that surgery alone yielded the best consequences for EMP when negative surgical margins were obtained [7]. Combined therapy is suggested when complete surgical tumor resection can not be applied and/or lymph node areas are effected. Meanwhile, these are the results of retrospective studies and require confirmation by prospective randomized trials.

### 4.3. Chemotherapy

In most series, adjuvant chemotherapy (CHT) has no beneficial effect on disease control or prevention of progression to multiple myeloma [40, 54]. A survival advantage was stated in a study that compared adjuvant melphalan and prednisone given for three years after RT with “RT alone”. Nevertheless, the study concerned included a small number of patients [55]. For the patients with tumors larger than 5 cm and high-grade histology, adjuvant CHT may be considered [29]. CHT may also be considered to unresponded patients to RT. Treatment schedules which are effective against multiple myeloma can be considered for these patients [29]. Holland et al. showed that CHT delays the progression time of plasmacytoma to MM. Nevertheless, its use did not decrease the conversion rate [21]. Besides, after progression to MM, the patients, who received CHT, had the same survival time as those patients who did not receive CHT [21]. Furthermore, it is suggested that early exposure to CHT may speed up the progression of resistant subclones and, therefore, limit later therapeutic options, when they may be more beneficial [1]. In addition, in one series, secondary leukemia developed in 4 of 7 patients with SBP who received adjuvant melphalan-based CHT after RT had been completed [56].

## 5. Followup and Further Outpatient Care

Patients are reevaluated with the measurements of M-protein and complete blood counts for progression and development of MM. It should be repeated at 6-week intervals for the first 6 months and then with prolongation of clinic visits. If a new bone pain takes place, additional workup like appropriate imaging will be needed [57].

## 6. Conclusions

The most common pattern of recurrence is not local relapse but systemic myeloma progression, being the main problem for the prognosis of the disease. Moreover, progression to MM may probably be related with factors such as age rather than local treatment.

The diagnosis and staging of plasmacytoma need an evaluation with more specific histological, phenotypic, and radiographic methods in order to exclude occult MM and other plasma cell neoplasm. Also, there is a need for further prospective studies with large series evaluating SBP and EMP separately to elucidate the argument on prognostic factors and treatment options of the disease. Close cooperative work done by a team of a hematologist, a radiotherapist, and a surgeon will ensure the best results after the treatment.

## Figures and Tables

**Table 1:** Solitary plasmacytoma of bones: representative treatment results.

Author	*n*	f/u	LC (%)	PMM (%)	OAS (%)
Wilder et al. [35]	60	94 mo	90	62	59
Knobel et al. [25]	206	56 mo	79	51	50
Tsang et al. [32]	32	95 mo	87	64	65
Kilciksız et al. [24]	57	**2.4 **y	94	4.1 y	68
Frassica et al. [23]	46	90	89	54	45
Bataille and Sany [33]	114	>10 y	88	58	68
Galieni et al. [40]	32	69 mo	91	68	49

mo: months, y: years f/u: Median followup, LC: Local control (10-year rate), PMM: progression to myeloma (10-year rate), and OAS: over all survival (10- year rate).

**Table 2:** Solitary Extramedullary Plasmacytoma: Representative Treatment Results.

Author	*n*	f/u	LC (%)	PMM (%)	OAS (%)
Kilciksiz et al. [24]	23	**2.4 **y	95	7.4 y	89
Ozsahin et al. [3]	52	56	74	36	72
Galieni et al. [40]	46	118	92	15	78 (15 y)
Tournier-Rangeard [42]	17	80.5	88.2	63.8	63.4
Strojan et al. [43]	26	61	87	8	61
Leibross et al. [44]	22	—	95	32	56
Chao et al. [45]	16	66	100	31	54
